# Solution-processed thickness engineering of tellurene for field-effect transistors and polarized infrared photodetectors

**DOI:** 10.3389/fchem.2022.1046010

**Published:** 2022-10-13

**Authors:** Fangfang Chen, Dingwen Cao, Juanjuan Li, Yong Yan, Di Wu, Cheng Zhang, Lenan Gao, Zhaowei Guo, Shihong Ma, Huihui Yu, Pei Lin

**Affiliations:** ^1^ School of Physics and Microelectronics, Key Laboratory of Materials Physics of Ministry of Education, Zhengzhou University, Zhengzhou, China; ^2^ School of Physics, Henan Normal University, XinXiang, China; ^3^ School of Materials Science and Engineering, National Joint Engineering Research Center for Abrasion Control and Molding of Metal Materials, Henan University of Science and Technology, Luoyang, China; ^4^ Academy for Advanced Interdisciplinary Science and Technology, Beijing Advanced Innovation Center for Materials Genome Engineering, University of Science and Technology Beijing, Beijing, China

**Keywords:** tellurene, oxidative etching, thickness engineering, field-effect transistors, polarizd infrared photodetection

## Abstract

Research on elemental 2D materials has been experiencing a renaissance in the past few years. Of particular interest is tellurium (Te), which possesses many exceptional properties for nanoelectronics, photonics, and beyond. Nevertheless, the lack of a scalable approach for the thickness engineering and the local properties modulation remains a major obstacle to unleashing its full device potential. Herein, a solution-processed oxidative etching strategy for post-growth thickness engineering is proposed by leveraging the moderate chemical reactivity of Te. Large-area ultrathin nanosheets with well-preserved morphologies could be readily obtained with appropriate oxidizing agents, such as HNO_2_, H_2_O_2_, and KMnO_4_. Compared with the conventional physical thinning approaches, this method exhibits critical merits of high efficiency, easy scalability, and the capability of site-specific thickness patterning. The thickness reduction leads to substantially improved gate tunability of field-effect transistors with an enhanced current switching ratio of ∼10^3^, promoting the applications of Te in future logic electronics. The response spectrum of Te phototransistors covers the full range of short-wave infrared wavelength (1–3 μm), and the room-temperature responsivity and detectivity reach 0.96 AW^-1^ and 2.2 × 10^9^ Jones at the telecom wavelength of 1.55 μm, together with a favorable photocurrent anisotropic ratio of ∼2.9. Our study offers a new approach to tackling the thickness engineering issue for solution-grown Te, which could help realize the full device potential of this emerging p-type 2D material.

## Introduction

Monoelemental two-dimensional (2D) crystals represent a particular category in the 2D materials family because of their unique physicochemical properties (Mannix et al., 2017). Graphene is indisputably the most famous one that has triggered the whole research field. Nevertheless, research on compound 2D materials (e.g., transition metal disulfides, metal halides, and transition metal carbides/nitrides) seems to dominate this area owing to their much larger numbers. With the substantial progress in synthesis technologies, a series of 2D main group elements and transition metals have been experimentally realized over the past few years and reawaken the researcher’s intense interest (Glavin et al., 2020; Fan et al., 2021; Si et al., 2021; Zhou et al., 2021). The elemental 2D materials typically exhibit metallic or semiconducting characteristics with high room-temperature carrier mobility, strong light absorption, and emerging topological properties, enabling potential widespread applications in future electronics, photonics, and energy-related devices (Nevalaita and Koskinen, 2018; Lin et al., 2020; Wu Z et al., 2021; Bat-Erdene et al., 2022). Moreover, the elemental materials bear a distinct advantage over 2D compounds in high-quality synthesis because the dissociation and stoichiometric problems do not exist.

Of particular interest among them is the emerging 2D tellurium (Te) that exhibits an intrinsic p-type transport characteristic and high hole mobility of ∼1,300 cm^2^·V^−1^·s^−1^, offering a new material platform to combine with many n-type 2D crystals for van der Waals (vdW) heterojunction electronics (Huang et al., 2017; Zhu et al., 2017; Wang et al., 2018; Tong et al., 2020; Zhao et al., 2021; Zhang et al., 2022) Besides, the highly-tunable bandgap of Te (∼0.35–1.2 eV) perfectly bridges the gap between semimetals and the widely-studied 2D transition metal disulfides and has unique applications in the short-wave infrared (SWIR) photodetection (Amani et al., 2018; Deckoff-Jones et al., 2019; Chaves et al., 2020; Peng et al., 2021; Ma et al., 2022). In particular, its strong anisotropic lattice structure enables the direct detection of the polarization information of infrared light, which is difficult to realize with the current infrared imaging technology. Generally, Te shares many similar properties with black phosphorus (BP) but has better ambient stability due to its higher oxidation barrier in ambient. Many previous studies have established that the performance of Te-based transistors and detectors hardly degrades over several months, even without any encapsulation (Wang et al., 2018; Tong et al., 2020; Ma et al., 2022).

Despite the versatile synthesis techniques for Te, such as chemical vapor deposition (Huang et al., 2021; Peng et al., 2021; Yang et al., 2022; Zhao et al., 2022), physical vapor deposition (Wang et al., 2014; Tao et al., 2021), and molecular beam epitaxy (Chen et al., 2017; Zheng W et al., 2021), the substrate-free solution approach offers distinct advantages of low temperature (<200°C) and scalability. It has been widely used to prepare monocrystal quasi-2D nanosheets with a lateral size of over 100 μm (Yao et al., 2021a). Nevertheless, due to its special growth mechanism, this method could not easily acquire ultrathin Te by adjusting the synthesis parameters (Londono-Calderon et al., 2020; Li et al., 2021). The hydrothermally-processed 2D crystals using Na_2_TeO_3_ and polyvinylpyrrolidone (PVP) as the precursor possess a typical thickness larger than 20 nm, while the Te flakes synthesized by oxidizing tellurium sodium hydride generally have a thickness of 50–170 nm (Wang et al., 2019; Hu et al., 2021). Due to the small bandgap and large carrier density, the thick Te usually exhibits degenerate charge transfer characteristics and a weak gate tuning of drain current at room temperature, limiting its application in logic electronics and optoelectronics.

Te is located between semiconducting selenium and metallic polonium in group VIA of the periodic table. The special electronic configuration endows it with moderate reactivity, allowing properties manipulation by appropriate chemical transformation (Xu et al., 2015). Leveraging this unique characteristic, we present a novel solution-processed oxidative etching method for Te post-growth thickness engineering. Scalable ultrathin Te nanosheets with well-retained morphologies could be easily obtained with appropriate oxidizing agents, such as HNO_2_, H_2_O_2_, and KMnO_4_. Particularly, thickness patterning is realized when combined with the photolithography technique, which enables the local modulation of material properties and the artificial design of novel device architectures. Field-effect transistors (FETs) fabricated with thinned Te reveals a much-enhanced current ON/OFF ratio of ∼10^3^, promoting their future applications in logic devices. The response spectrum of Te phototransistors covers the full range of SWIR wavelength (*λ* = 1–3 μm), and the responsivity and detectivity reach 0.96 AW^−1^ and 2.2 × 10^9^ Jones at the optical communication wavelength of 1.55 μm. Besides, a high photocurrent anisotropic ratio of ∼2.9 is obtained, which ensures the promising application of Te in polarized infrared imaging. Scalable Thickness engineering has been considered a major obstacle for solution-grown Te. Our study offers a new approach to tackling this issue, helping unleash the full device potential of this emerging 2D material.

## Materials and methods

### Hydrothermal synthesis of Te and post-growth thinning process

The synthesis of quasi-2D Te nanosheets starts with dissolving 0.6 g of PVP (*Mw* 58,000) and 0.11 g of Na_2_TeO_3_ in 16 ml of ultrapure water. The mixture was magnetically stirred for 20 min to form a transparent homogeneous solution. 2 ml of ammonia solution (25%–28%, w/w) and 1 ml of hydrazine hydrate (80%, w/w) were then added. After another 1 min of magnetic stirring, the nutrient solution was transferred into a 50 ml hydrothermal reactor. The crystal growth process was performed at 180°C for 4.5 h. The resulting Te nanosheets were centrifuge-washed several times at 5,000 rpm and finally redispersed in the ultrapure water.

An oxidative-etching method with HNO_2_ was first adopted for post-growth thinning of Te nanosheets. The oxidant solution was prepared by adding 25 mg of sodium nitrite (NaNO_2_) into 40 ml of dilute hydrochloric acid (HCl, 10 mmol/L). The number of moles of H^+^ is slightly larger than that of NO^2-^ for the subsequent acid etching process. The thinning was performed by immersing drop-casted Te nanosheets on SiO_2_/Si substrate into the solution at room temperature (or mixing the oxidant solution with Te redispersion solution). Thickness engineering with other appropriate oxidizing agents, such as H_2_O_2_ and KMnO_4_, and the H_2_O_2_ concentration-dependent thinning effect were also investigated.

### Device fabrication

The FETs and phototransistors were fabricated by transferring Te nanosheets onto the SiO_2_/p^+^-Si substrate *via* a poly (vinyl chloride)-assisted dry transfer method (Wakafuji et al., 2020). The standard electron-beam lithography process was performed to define the source and drain patterns. High-work-function metals Pd/Au (10 nm/50 nm) were evaporated as the contact electrodes.

### Material and device characterizations

An optical microscope (SOPTOP-CX40M) was employed to investigate the Te morphologies. The accurate thickness of materials was measured with an atomic force microscope (AFM, Bruker dimension Icon). The transmission electron microscopy (TEM) characterization was performed with a JEM-2100 electron microscope operated at 200 kV. The polarized Raman spectra were taken using the LabRAM HR Evolution Raman system with a 532 nm excitation laser. The room-temperature electrical properties were acquired with a Keithley 4200A-SCS semiconductor parameter analyzer. Power-tunable lasers (980 nm, 1.55 μm, and 3.0 μm wavelength) coupled with optical fiber were used as the illumination source.

## Results and discussion

**FIGURE 1:**
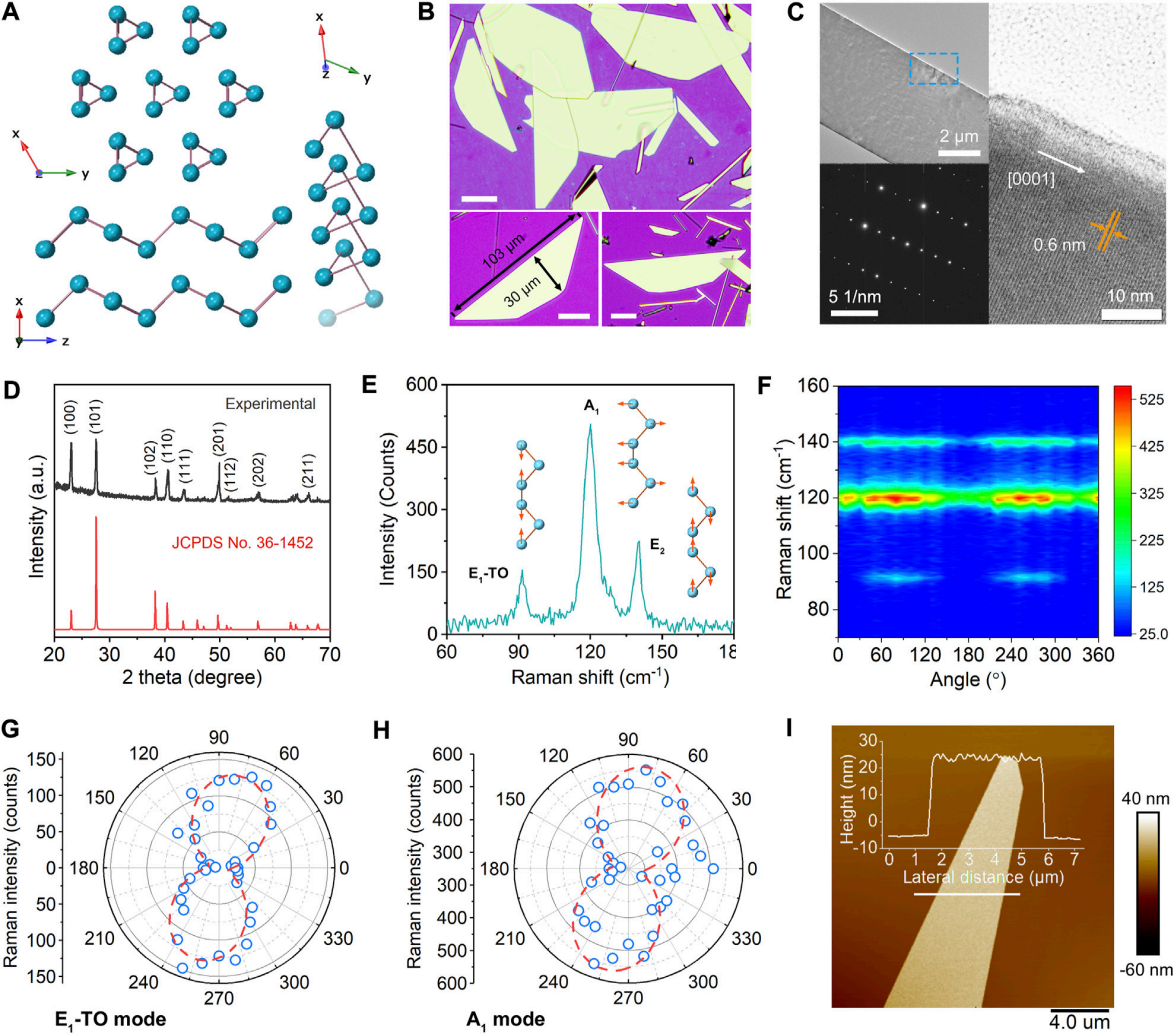
Characterizations of the solution-grown 2D Te flakes. **(A)** Schematic lattice structure of trigonal Te. **(B)** Optical images of Te nanosheets. The scale bar is 20 μm. **(C)** Low- and high-magnification TEM images of individual Te and corresponding selected area diffraction pattern, evidencing its single crystallinity and growth direction along the c-axis. **(D)** XRD pattern of synthetic Te. **(E)** Raman spectrum that shows characteristic peaks of E_1_-TO, A_1_, and E_2_ vibrational modes. **(F)** Angle-resolved polarized Raman mapping of Te nanosheet. Polar plots of Raman intensity for **(G)** E_1_-TO and **(H)** A_1_ mode. **(I)** AFM image of a typical Te nanosheet, the thickness is ∼30 nm.

The elemental Te typically forms a trigonal lattice structure, which consists of helical chains arranged hexagonally, as shown in Figure 1A (Agapito et al., 2013; Zhu et al., 2017; Qiao et al., 2018). The bonds between neighboring Te atoms on the same chain are covalent, whereas the interaction between chains is considered a mixture of electronic and vdW types (Weidmann and Anderson, 1971). Figure 1B exhibits the optical microscope images of solution-grown Te nanoflakes. It can be found that the length and width could exceed 100 and 30 μm, respectively. The Te crystal structure is determined using TEM and X-ray diffraction (XRD). Figure 1C presents the low- and high-magnification TEM images of an individual Te and corresponding selected area electron diffraction pattern with sharp spots, revealing the high crystallinity of synthetic nanosheets. The calculated interplanar distance of 0.6 nm is consistent with the trigonal-Te (0001) planes, which verifies that the length’s growth direction is *c*-axis oriented (Wang et al., 2018). The XRD result in Figure 1D matches well with the standard diffraction file JCPDS card (No. 36-1452), further confirming the trigonal structure of Te. The Raman spectrum in Figure 1E shows three sharp characteristic peaks at 91.5 cm^−1^, 119.9 cm^−1^, and 139.8 cm^−1^, corresponding to the E_1_-TO, A_1_, and E_2_ vibrational modes (Pine and Dresselhaus, 1971; Tong et al., 2020; Wang et al., 2022). Lorentzian fitting of these peaks yields a narrow full width at half maximum of 3.6 cm^−1^, 6.7 cm^−1^, and 3.2 cm^−1^, respectively (Supplementary Figure S1). Moreover, angle-resolved Raman spectra were measured to investigate the optical anisotropy properties of Te, as shown in Figure 1F. The intensity anisotropic ratios for E_1_-TO and A_1_ vibrational mode (Figures 1G,H) reach 29.4 and 2.1, evidencing its great potential for applications in polarized infrared photodetection (Supplementary Figure S2). The atomic force microscope is employed to measure the material thickness, ranging from about 20 to 120 nm (Supplementary Figure S3). Figure 1I shows an AFM image of a typical Te nanosheet and corresponding cross-section height profile, revealing a thickness of ∼30 nm.

The Kelvin probe force microscope (KPFM) measurement was performed to investigate the work function of Te on conductive Au substrate (Supplementary Figure S4B). The result indicates that the average contact potential difference Δ*V*_CPD_ of Te nanosheet is ∼240 mV larger than Au. Considering the determined work function of ∼5.1 eV for Au, the work function of Te is calculated to be ∼4.86 eV. Moreover, ultraviolet photoelectron spectroscopy (UPS) was also measured (Supplementary Figure S4A). The estimated work function of as-grown Te is ∼4.85 eV, which is consistent with the KPFM results.

**FIGURE 2:**
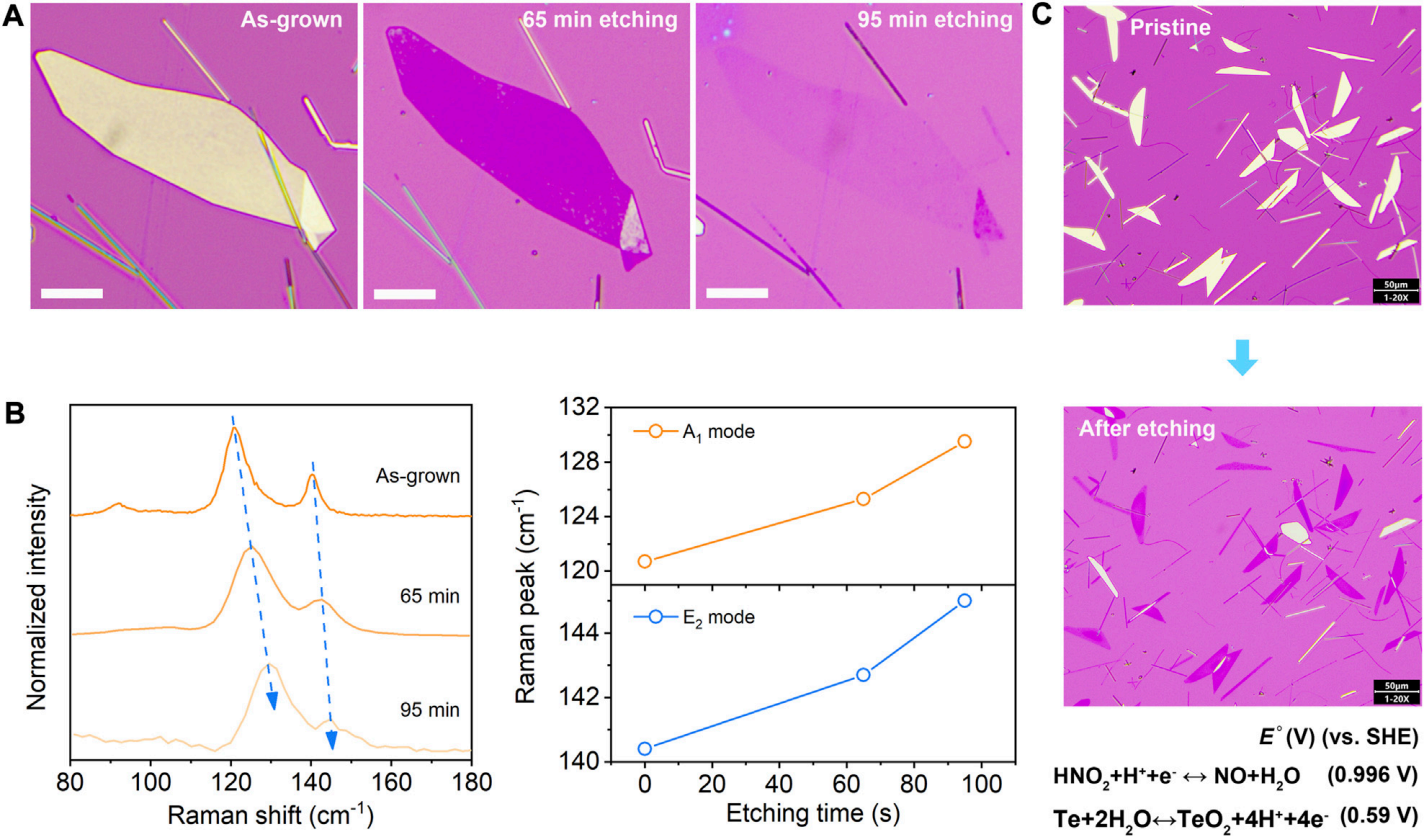
Controllable thinning of the Te nanosheets. **(A)** Optical images of an individual Te flake upon *in-situ* oxidative etching with HNO_2_ solution. Scale bar, 10 μm. **(B)** Corresponding Raman spectra of the Te sample, indicating blueshift of A_1_ and E_2_ peaks with thickness reduction. **(C)** Optical images of Te before and after the wet etching process with HNO_2_, revealing the capability of scalable thinning.

A major obstacle in the solution synthesis of Te is that the ultrathin nanosheets with a thickness less than 10 nm cannot be easily derived *via* tuning synthetic parameters (e.g., temperature, the mole ratio of precursors, synthesis time, etc.) (Wang et al., 2019; Hu et al., 2021). The large material thickness generally results in weak electrostatic tunability of the Te FETs and a high off-state current, which is unfavorable for logic electronics and optoelectronics (Wang et al., 2018; Zhao et al., 2020; Yao et al., 2021b). Despite the excellent ambient stability, the element Te has been demonstrated to possess moderate chemical reactivity (Yang et al., 2015; He et al., 2019). Using this unique characteristic, we propose a solution-processed wet-chemical strategy with the weak oxidizing agent HNO_2_ for the Te thickness engineering. The standard redox potential of HNO_2_/NO is ∼0.996 V (vs. standard hydrogen electrode), higher than that of TeO_2_/Te (0.59 V) (Bockris and Oldfield, 1955; Yang et al., 2018; Zheng B et al., 2021). Therefore, the elemental Te could be oxidized in dilute HNO_2_ solution, with the generated TeO_2_ being subsequently etched away by the excess H^+^, leading to the gradual reduction of material thickness. Figure 2A presents the optical images of an individual Te flake upon *in-situ* thinning with HNO_2_ solution for different times, showing an apparent gradual thickness reduction. From the Raman spectra in Figure 2B, it can be found that the A_1_ and E_2_ vibrational peaks blueshift with decreasing thickness, which agrees with our previous reports (Yao et al., 2021b). Moreover, the control experiments were carried out with individual NaNO_2_ and HCl solutions (Supplementary Figure S5). No apparent morphology change could be observed, confirming the thinning effect with HNO_2_.

Compared with the physical thinning approaches such as plasma, focused ion beam, or laser etching (Castellanos-Gomez et al., 2012; Wang et al., 2016; Nan et al., 2019), this method has a distinct advantage of low cost and easy scalability. The large-area ultrathin Te nanosheets could be easily prepared, as shown in Figure 2C. Based on the same rationale, other appropriate oxidizing agents such as H_2_O_2_ and KMnO_4_ are also employed for the thinning process, as demonstrated in Supplementary Figure S6. Ultrathin Te nanosheets with well-retained morphologies and controllable thickness are obtained, revealing the general applicability of this method. It should be noted that under the similar oxidant concentration, the thinning efficiency of H_2_O_2_ and KMnO_4_ is typically higher than HNO_2_, which is due to the higher standard redox potential. The H_2_O_2_ concentration-dependent thinning effect was also explored, and the details are presented in Supplementary Figure S7. Compared with the previously reported post-growth thinning of Te in mixed alkaline and acetone solution (Wang et al., 2018), our approach relies on a different mechanism. Furthermore, it is supposed to have higher thinning efficiency. Ultrathin Te crystals with a thickness smaller than 10 nm could be readily derived within several minutes.

**FIGURE 3:**
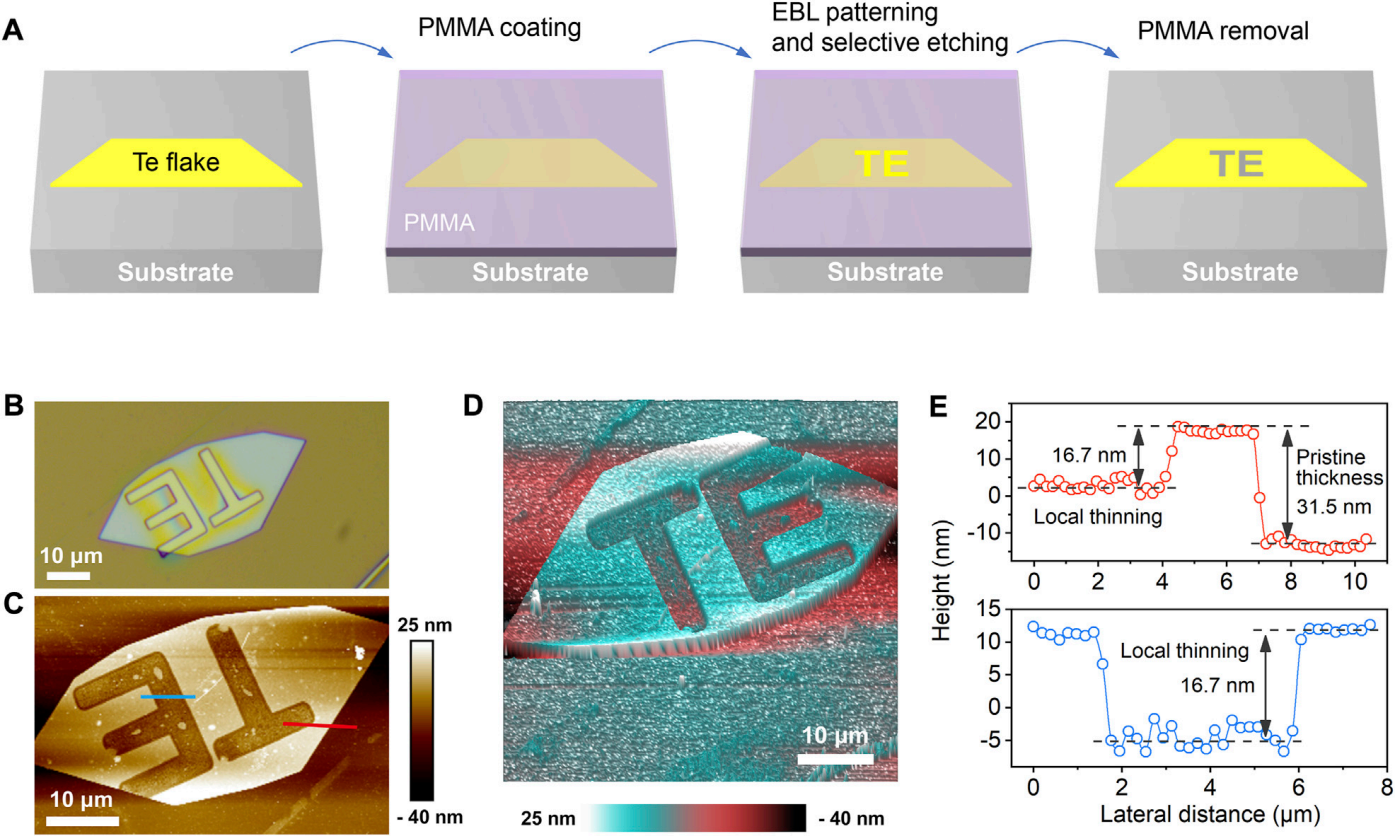
**(A)** Schematic fabrication process for the thickness patterning of Te nanosheet. **(B)** Optical image of the Te nanosheet with a “TE” photoresist pattern on the surface. **(C–D)** 2D and 3D AFM images of the locally-thinned Te flake. **(E)** Height profiles along the blue and red lines in **(C)**, revealing the capability of local thinning with this oxidative etching method.

Additionally, due to the mild processing conditions, this approach enables arbitrary site-specific thickness patterning when combined with the conventional photolithography technique (Figure 3A). Figure 3B presents the optical image of Te nanosheet with a “TE” poly (methyl methacrylate) pattern on the surface. The local thinning characteristic is confirmed from the atomic force microscope images after 30 min etching in HNO_2_, as shown in Figures 3C,D. Figure 3E shows the height profiles of pristine and thinned Te areas. The thickness of pristine Te is measured to be ∼31.5 nm, and the etching depth is ∼16.7 nm. Moreover, the etched areas exhibit relatively uniform thickness and sharp edges, revealing the potential of creating high-resolution patterns with this method. As is known, the electronic structure is strongly thickness-dependent for 2D materials. The artificial thickness patterning offers vast enticing possibilities to locally manipulate the physical properties of Te for designing novel electronics and optoelectronics.

**FIGURE 4:**
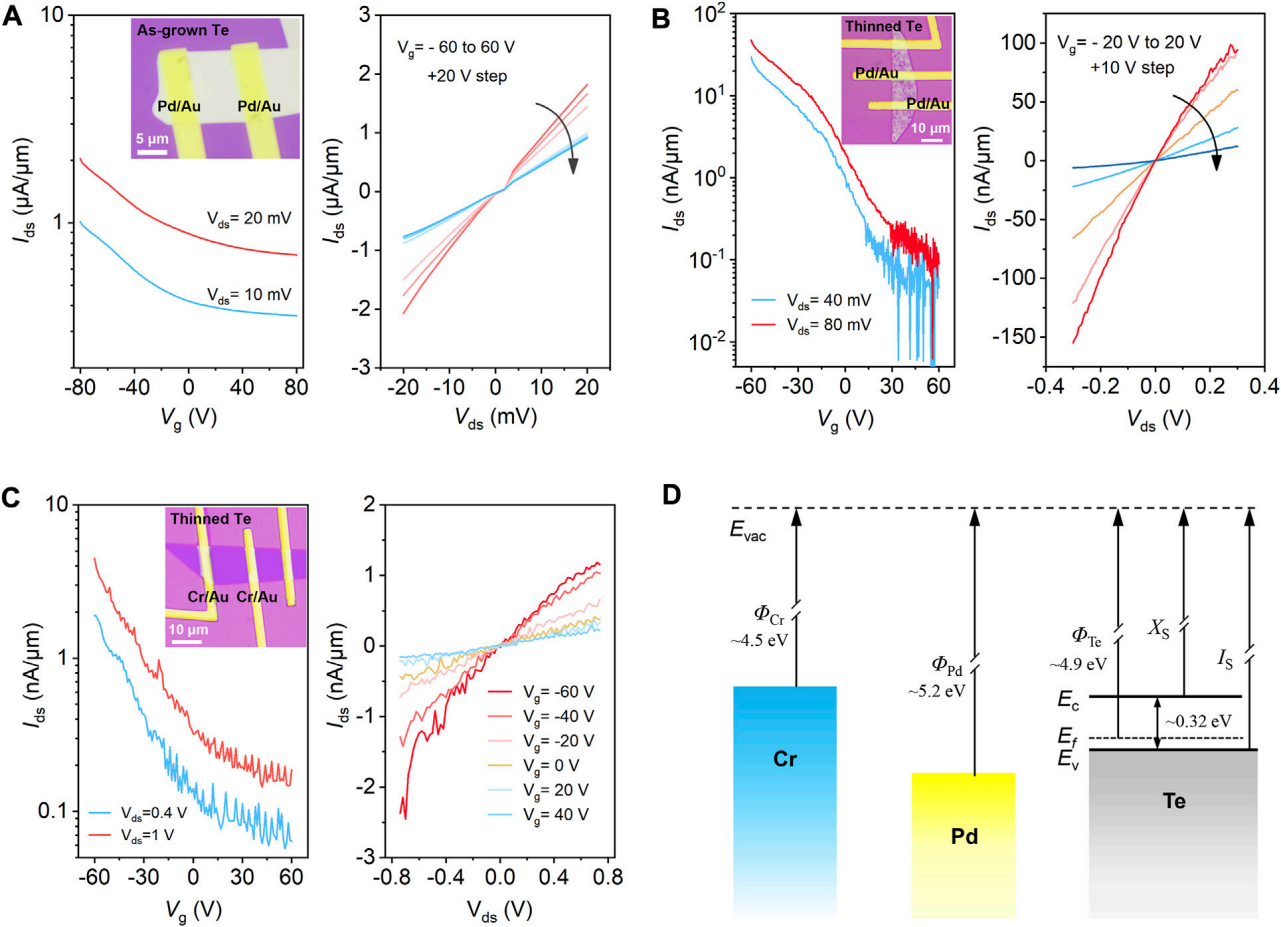
Characterizations of the pristine and thinned Te FETs. **(A)** Transfer and output curves of FET fabricated with as-grown Te nanosheet, and the inset shows the corresponding optical image of the device. **(B)** Transfer and output curves of FET fabricated with thinned Te. Inset shows the optical image of the device, and Pd/Au is employed as the source and drain electrodes. **(C)** Transfer and output curves of another thinned Te FET, in which Cr/Au is used as the electrode. **(D)** Schematic energy band profiles for Cr, Pd, and Te.

The thinning effect on the electrical properties of Te FETs is further explored. Figure 4A shows the transfer (*I*_ds_-*V*_g_) and output (*I*_ds_-*V*_ds_) curves of FET fabricated with as-grown Te nanosheet, revealing a typical p-type transport characteristic and favorable room-temperature hole mobility of ∼630 cm^2^·V^−1^·s^−1^ (Supplementary Figure S8). Nevertheless, the ON/OFF ratio is generally less than one order of magnitude, and gate control over the drain current is weak owing to the considerable material thickness and high carrier density. In contrast, the FET with thinned Te channel (Figure 4B) presents significantly improved electrostatic tunability, with the ON/OFF ratio reaching ∼10^3^ at *V*_g_ of ± 60 V. Moreover, the ratio is supposed to be further improved by optimizing the device structure, such as employing high-*k* dielectrics and performing surface passivation. The tunable electrical properties in Te and the feasibility of local thickness engineering provide a versatile approach to fabricating on-demand electronics and optoelectronics. The effect of contact electrodes on the FET properties is also investigated. Figure 4C displays the transfer and output curves of another FET with thinned Te channel and Cr/Au (10 nm/50 nm) electrodes. The thickness of Te is estimated to be thinner than that in Figure 4B from the optical contrast, which should logically present a higher ON/OFF switching ratio. However, the measured ON/OFF ratio is lower. Meanwhile, a more pronounced Schottky contact is observed from the output characteristics. One of the reasons may be that the work function of Cr (∼4.5 eV) is smaller than that of Pd (∼5.2 eV), as schematically displayed in Figure 4D. The resulting larger Schottky barrier height at the Cr/*p*-Te interface and, thus, large contact resistance restrains the hole transport capabilities (details are shown in Supplementary Figure S9).

**FIGURE 5:**
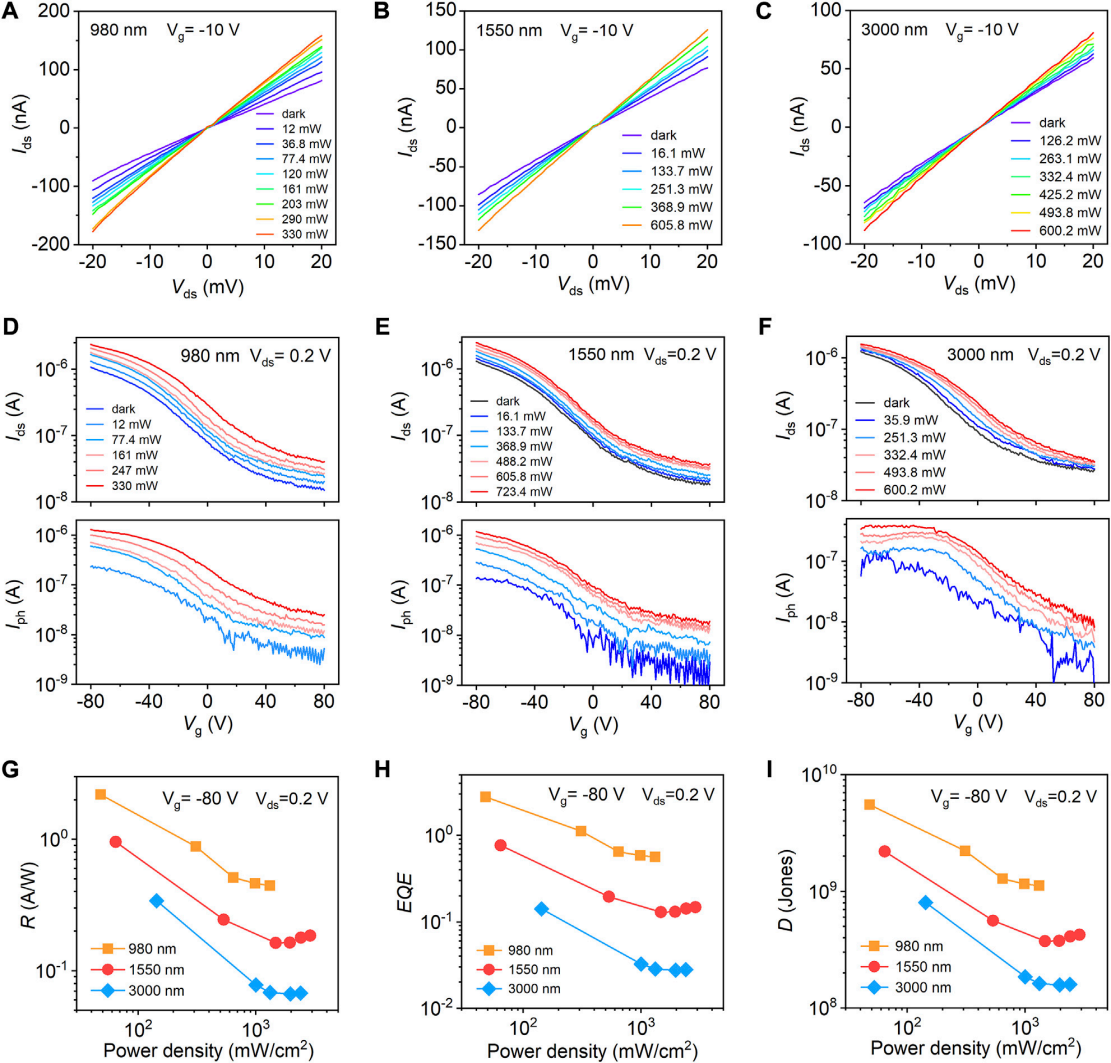
The *I*_ds_-*V*_ds_ curves of Te phototransistors under varying 980 nm **(A)**, 1,550 nm **(B)**, and 3,000 nm **(C)** illumination power. The gate voltage *V*_g_ is fixed at -10 V. **(D–F)** Transfer characteristics (upper part) and gate-dependent net photocurrent *I*_ph_ (lower part) under different illumination wavelengths. **(G–I)** Illumination power-dependent photoresponsivity (*R*), external quantum efficiency (*EQE*), and detectivity *D* for different wavelengths at *V*_g_ = −80 V and *V*_ds_ = 0.2 V.

Optoelectronic characteristics of the Te FETs were further studied to demonstrate their applications in photodetectors. Schematic and optical images of the device are shown in Supplementary Figure S10. Figures 5A–C presents the *I*_ds_-*V*_ds_ output curves (at *V*_g_ = −10 V) of Te phototransistors under the 980 nm, 1.55 μm, and 3.0 μm laser illumination with varying power densities, which demonstrate a broad response spectrum covering the entire SWIR wavelength (*λ* = 1–3 μm). The net photocurrent *I*_ph_ increases monolithically with increasing incident power for all wavelengths. In contrast, the responsivity (*R*) decreases with increasing light intensity, which may be attributed to the decreased density of unoccupied states in Te and enhanced photocarrier recombination under high optical power (Supplementary Figure S11) (Xue et al., 2016; Zeng et al., 2018; Zeng et al., 2020; Wu D et al., 2021). Besides, the photoresponse could be further effectively modulated with electrostatic gating. Figures 5D–F presents the transfer characteristics of the Te transistor (upper part) and corresponding gate-dependent *I*_ph_ (lower part) under different illumination wavelengths. Generally, the photocurrent and thus the *R* increase with negative gate voltage. For the 980 nm laser, the *R* reaches 2.2 AW^−1^ at an incident power density of 48 mWcm^−2^ at *V*_g_ of −80 V and *V*_ds_ of 0.2 V. For the optical communication wavelength of 1.55 μm, a responsivity of 0.96 AW^−1^ is obtained under 64.4 mWcm^−2^ illumination density, together with an external quantum efficiency (*EQE*) of 76.5% and detectivity (*D*) of 2.2 × 10^9^ Jones (Figures 5G–I). The calculation details of these critical parameters (*R*, *EQE*, and *D*) are shown in Supplementary Figure S12 and Supplementary Note S1. Compared with 980 nm, the reduced photoresponse properties for 1.55 and 3.0 μm light are due to the lower absorption coefficient of Te for longer wavelengths (Amani et al., 2018; Tong et al., 2020).

**FIGURE 6:**
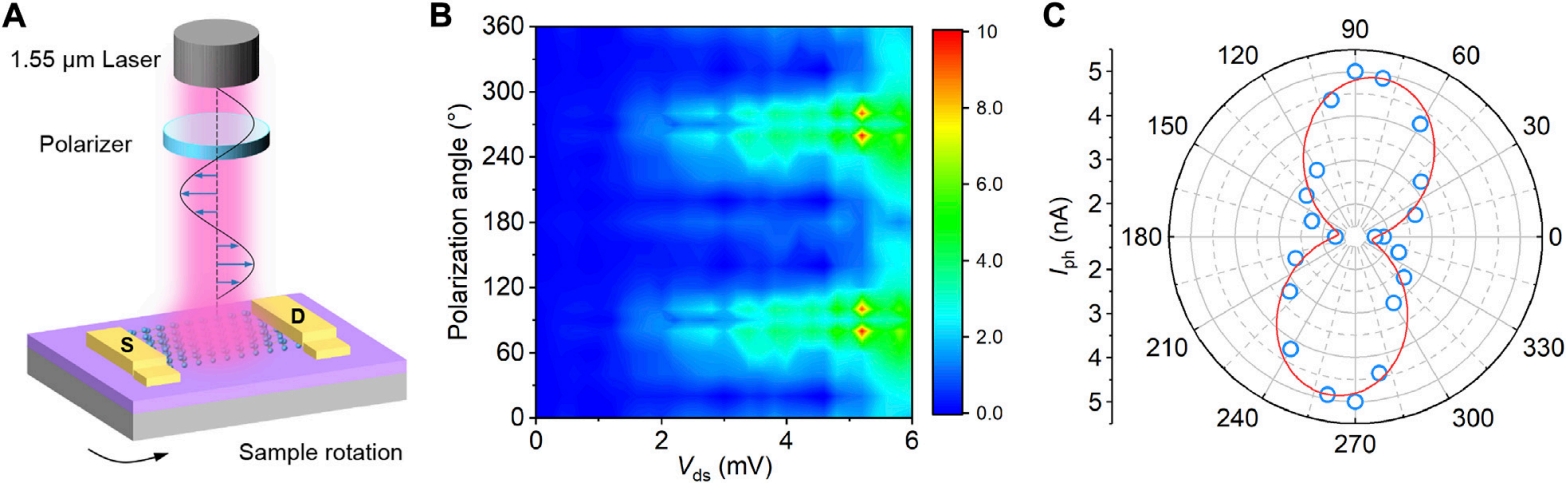
Polarized infrared photoresponse of the Te transistor under the incident wavelength of 1.55 μm. **(A)** Schematic experimental setup for measuring the polarization-resolved photoresponse. **(B)** Contour plot of polarized photoresponse characteristics of the Te phototransistor under constant 340 mW 1,550 nm illumination. **(C)** Polar plot of the *I*_ph_ as a function of the sample rotation angle, the *V*_ds_ is 6 mV.

Polarized Raman characterization in Figures 1F–H has demonstrated the favorable optical anisotropy of Te, revealing its feasibility for polarized SWIR photodetection. In light of this, the polarization-resolved photoresponse of Te phototransistor under 1.55 μm illumination was further investigated, with the experimental setup schematically shown in Figure 6A. The linear polarization direction of the laser and incident optical power is fixed during measurement, and the *I*_ds_-*V*_ds_ photoresponse curves are measured at different sample rotation angles. Figure 6B displays the contour map of polarized *I*_ph_-*V*_ds_ characteristics of the Te transistor, exhibiting obvious periodic variation with the sample rotation angles and changes periodically. The polar plot of *I*_ph_ as a function of sample rotation angle at *V*_ds_ of 6 mV is shown in Figure 6C. The corresponding photocurrent *I*_ph_-*V*_ds_ curves at each rotation angle are presented in Supplementary Figure S13. The anisotropic ratio, defined as the maximum *I*_ph_ over the minimum, reaches up to ∼2.9, which is very competitive in the SWIR band and comparable with other widely-studied 2D materials such as BP and PdSe_2_ (Yuan et al., 2015; Pi et al., 2021). The results suggest that the 2D Te can be a promising material platform for future polarized infrared imaging applications.

## Conclusion

In summary, we proposed a novel solution-processed strategy for the thickness engineering of 2D Te based on the oxidative-etching mechanism. The gradual material thickness reduction was readily achieved using appropriate oxidants, such as HNO_2_, H_2_O_2_, and KMnO_4_. Compared with the conventional physical thinning approaches, this method has several distinct advantages: Low cost, easy scalability, and facile processing conditions. In particular, site-specific thickness patterning was also realized in combination with the mature photolithography technique, which opens vast possibilities for arbitrarily tailoring the local properties of Te. The field-effect transistors fabricated with thinned Te present a significantly improved gate-tunability, thus promoting their applications in future logic devices. For optoelectronic applications, the response spectrum of Te phototransistors covers the whole SWIR band (*λ* = 1–3 μm). A favorable responsivity of 0.96 AW^−1^ and detectivity of 2.2 × 10^9^ Jones was obtained at the optical communication wavelength of 1.55 μm, together with an anisotropic photocurrent ratio of ∼2.9. As the thickness engineering approach is implemented by leveraging the material’s intrinsic chemical reactivity, it should apply to 2D Te prepared by other techniques, such as physical vapor deposition, chemical vapor deposition, or thermal deposition. Meanwhile, the feasibility of site-specific thinning may inspire the design of more sophisticated Te-based electronics and optoelectronics, which help realize its full device potential.

## Data Availability

The original contributions presented in the study are included in the article/Supplementary Material, further inquiries can be directed to the corresponding authors.
