# Supplementary material for: Solution-processed thickness engineering of tellurene for field-effect transistors and polarized infrared photodetectors
Source: Front Chem. 2022 Oct 13;10:1046010. doi: 10.3389/fchem.2022.1046010 (PMC9606353; doi:10.3389/fchem.2022.1046010)
Supplement: Supplementary file 1 [file DataSheet1.docx]

Supplementary Material


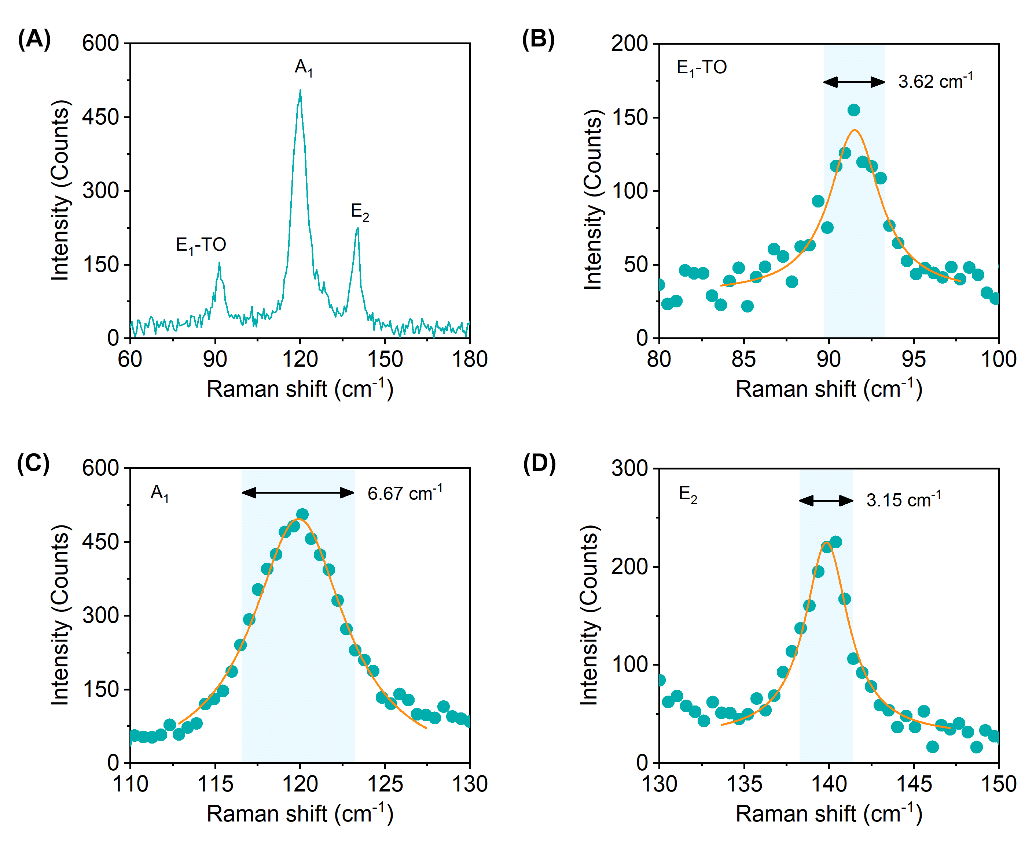


**Supplementary Figure S1.** Three characteristic peaks at 91.5 cm^-1^, 119.9 cm^-1^, and 139.85 cm^-1^ could be observed from the Raman spectrum in **(A)**, corresponding to the E_1_-TO, A_1_, and E_2_ vibration modes. The peaks are fitted with a Lorentz function, obtaining a narrow full-width-half-maximum (FWHM) of 3.62 cm^-1^ **(B)**, 6.67 cm^-1^ **(C)**, and 3.15 cm^-1^ **(D)**, respectively, indicating the high crystallinity of as-grown Te nanosheets.


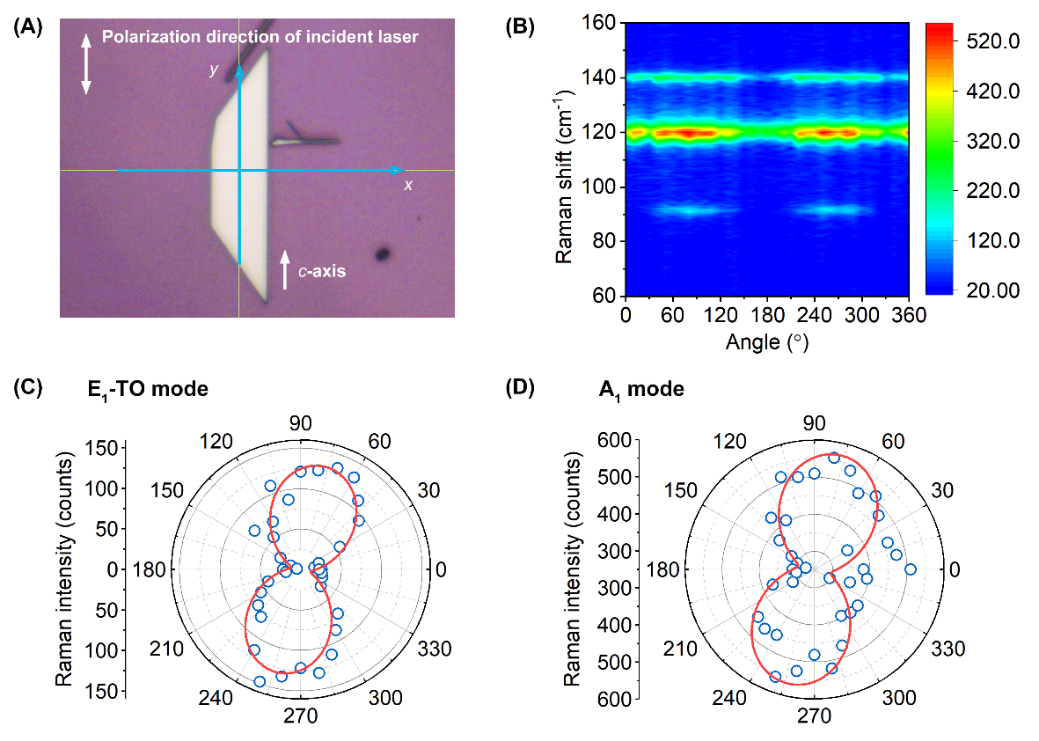


**Supplementary Figure S2.** Angle-resolved polarized Raman characterization of Te nanosheet. **(A)** Optical image of the Te nanosheet. **(B)** Polarized Raman mapping of the Te sample. **(C-D)** Polar plots of the Raman intensity for E_1_-TO and A_1_ mode.

For angle-resolved Raman measurements, the polarization direction of the incident 532 nm laser was modulated to be parallel to the *c*-axis of the Te nanosheet. The Raman spectra were obtained by clockwise rotating substrate with a step of 10º. The Raman intensity anisotropic ratios for E_1_-TO and A_1_ vibrational mode reach 29.4 and 2.1, respectively, evidencing the high crystallinity of Te and its potential applications in polarized infrared light detection.


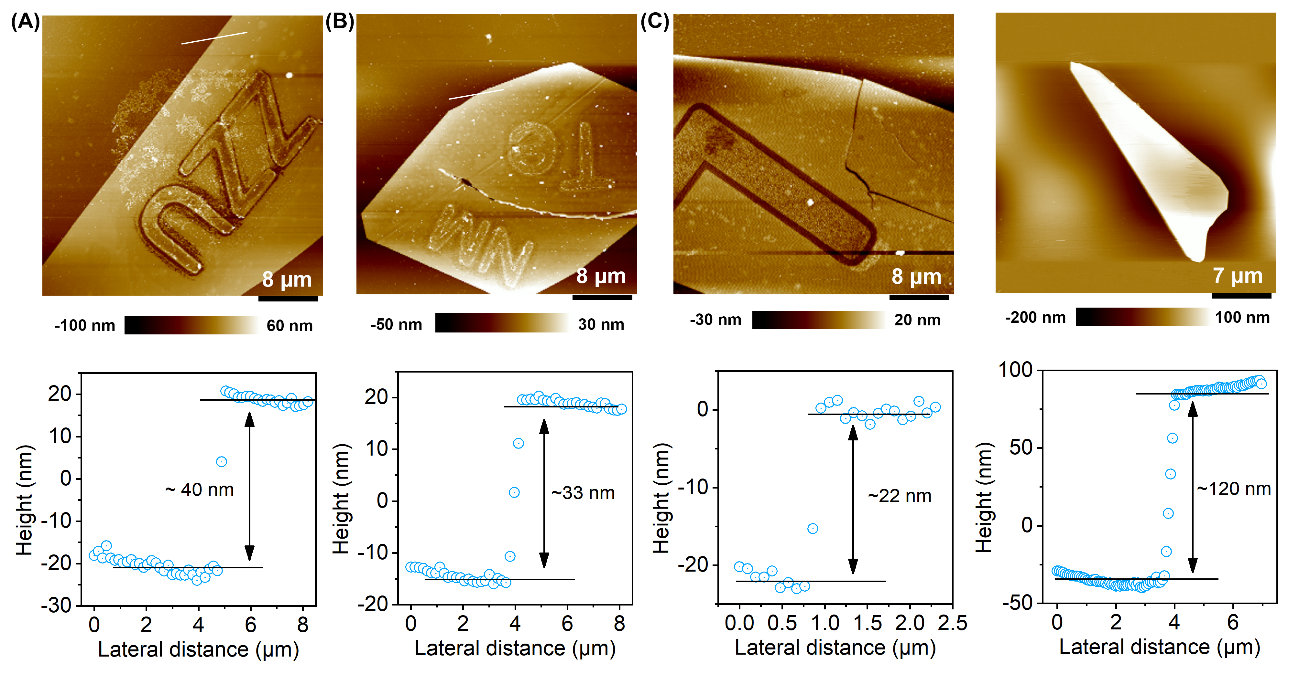


**Supplementary Figure S3.** AFM characterizations of as-grown Te nanosheets, revealing a thickness distribution from 20 to 120 nm.


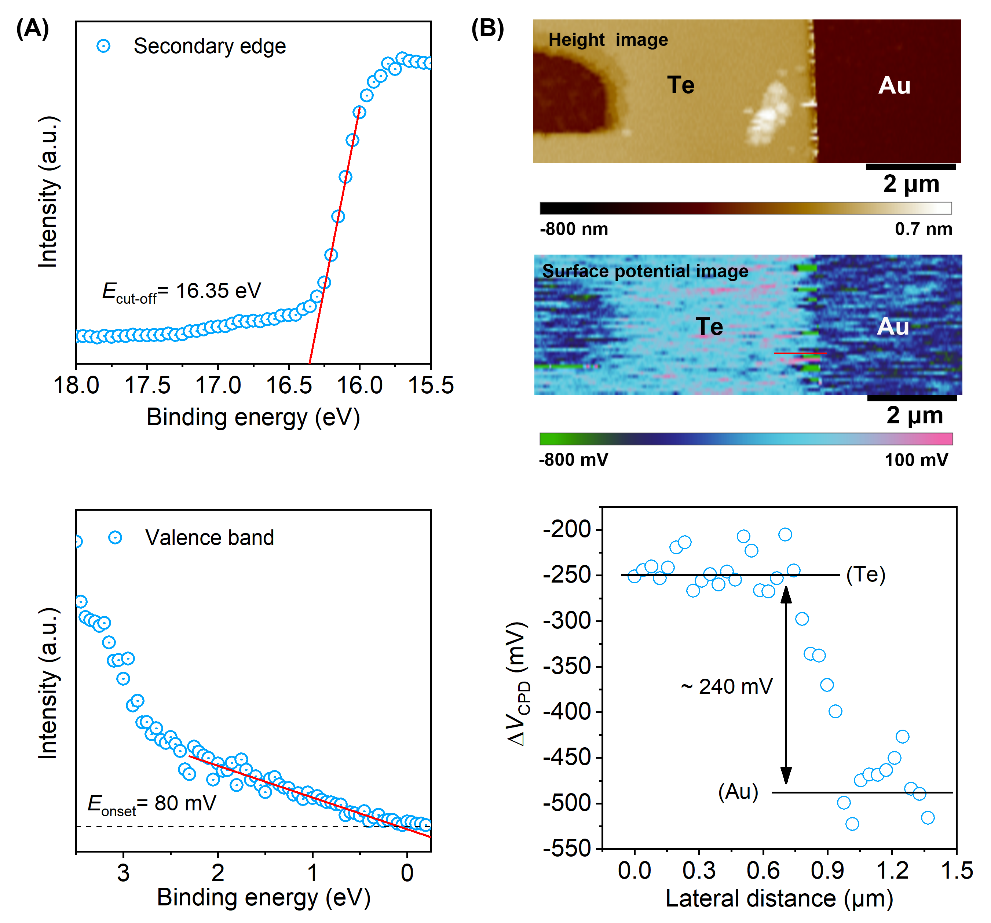


**Supplementary Figure S4.** Ultraviolet photoelectron spectroscopy **(**UPS) **(A)** and Kelvin probe force microscopy (KPFM) **(B)** measurements of Te nanosheets. A surface potential profile is illustrated in the lower part of **(B)**, revealing that the average contact potential difference Δ*V*_CPD_ of Te nanosheet is ~240 mV larger than Au. Considering the determined work function of ~5.1 eV for Au, the work function of Te is calculated to be ~4.86 eV. The UPS measurements indicate that the work function of Te is about 4.85 eV (=21.2 eV-16.35 eV), consistent with the KPFM result.


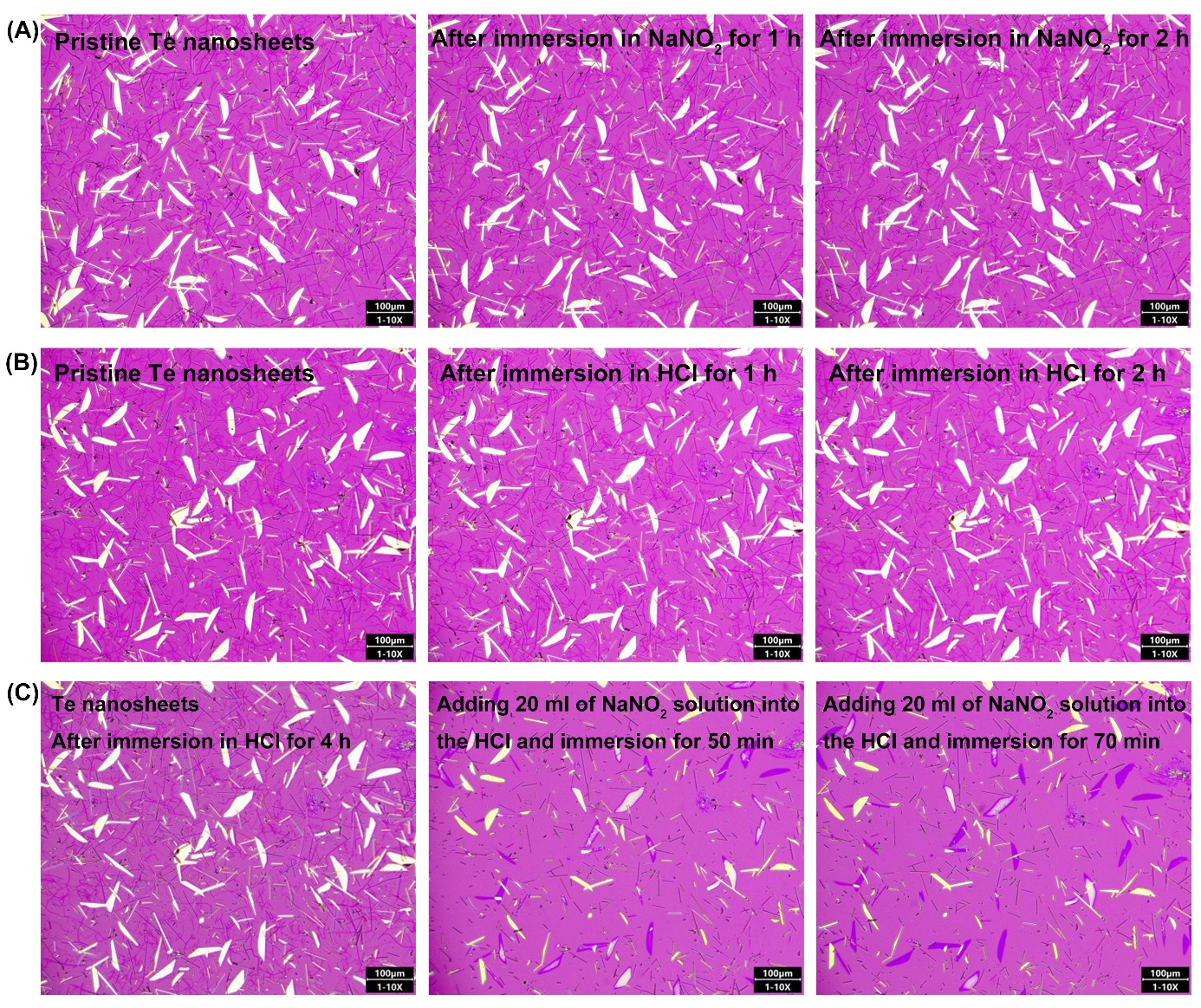


**Supplementary Figure S5.** Control experiments that verify the thinning effect of Te nanosheets with HNO_2_ solution. **(A)** Left: pristine (as-grown) Te nanosheets; Middle: after immersion in 20 ml of NaNO_2_ solution (0.72 mol/L) for 1 h; Right: after immersion in the NaNO_2_ solution for 2 h, showing no apparent morphology change. **(B)** Left: pristine Te flakes on SiO_2_/Si substrate; Middle: after immersion in 15 ml of HCl solution (0.05 mol/L) for 1 h; Right: after immersion in the HCl solution for 2 h. No apparent morphology change could be observed, indicating the excellent stability of Te nanosheets even in dilute acid solution. **(C)** Left: Te nanosheets after immersion in 15 ml of HCl solution (0.05 mol/L) for 4 h; Middle: adding 20 ml of NaNO_2_ solution (0.72 mol/L) into the HCl solution and immerse the Te flakes for 50 min; Right: immersion of Te flakes in the mixed solution for 70 min, from which the large-area thinned Te nanosheets could be observed. On the one hand, the results indicate that Te nanosheets possess good stability in neutral and even acid solutions. On the other hand, it verifies the thinning effect of HNO_2_ rather than the individual H^+^ or NO^2-^.


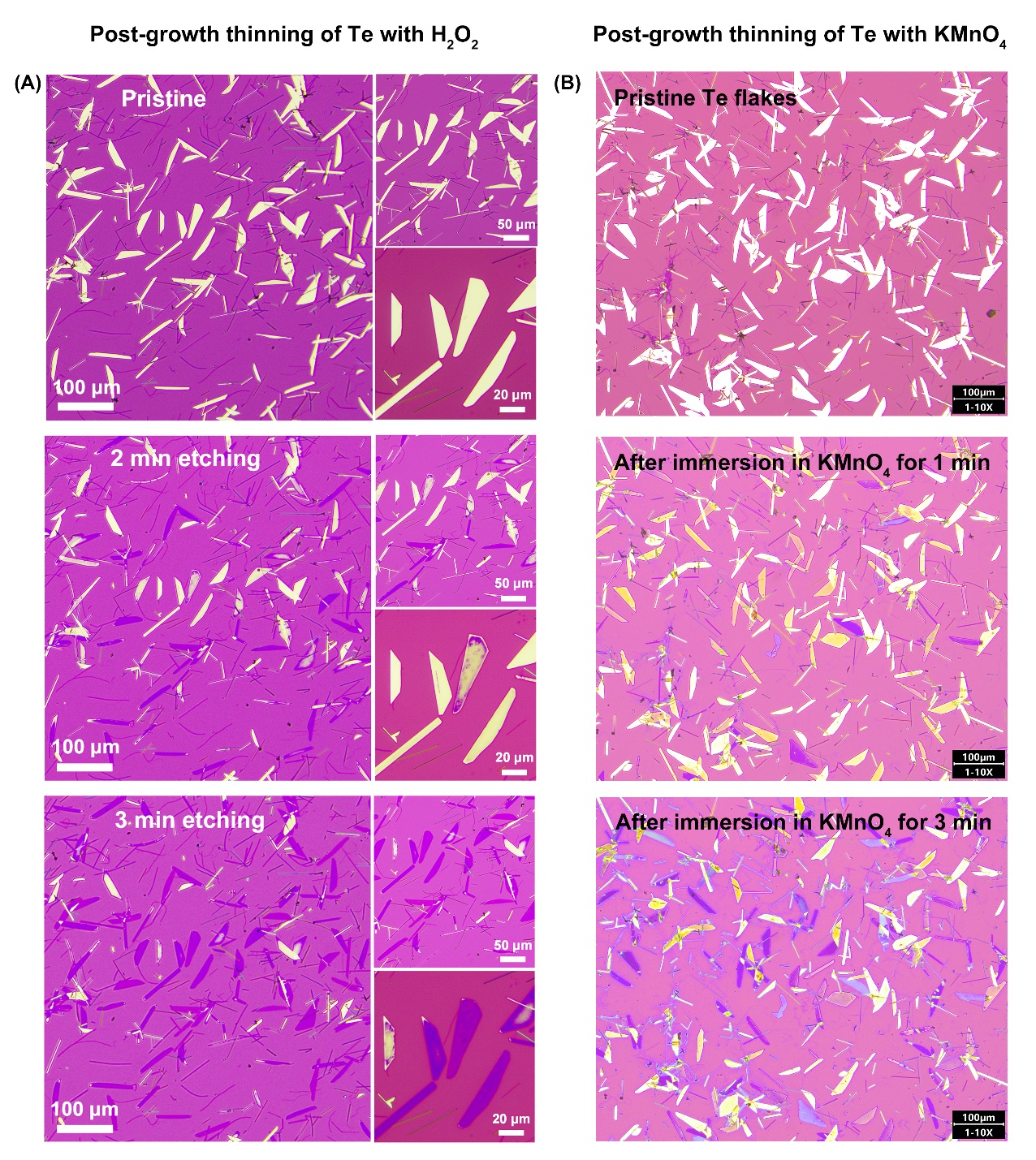


**Supplementary Figure S6.** Scalable thinning of Te nanosheets with **(A)** H_2_O_2_ and **(B)** KMnO_4_ solution. The standard redox potential of H_2_O_2_/H_2_O (H_2_O_2_+2H^+^+2*e*^-^ ⇌ 2H_2_O) and KMnO_4_ (MnO^4-^+8H^+^+5*e*^-^ ⇌ Mn^2+^+4H_2_O) is 1.776 V and 1.51 V, higher than TeO_2_/Te. The KMnO_4_ solution is prepared by mixing 10 mg of KMnO_4_ with 400 ml of dilute HCl (~30 mmol/L). The H_2_O_2_ (30%) is diluted by adding 1 ml of H_2_O_2_ into 1000 ml of deionized water. The as-grown Te nanosheets are drop-cast on the SiO_2_/Si substrate and then immersed into the solutions for thinning process at room temperature. Particularly, due to the higher standard redox potential of H_2_O_2_/H_2_O and KMnO_4_ than HNO_2_/NO (HNO_2_+H^+^+*e*^-^ ⇌ NO+H_2_O *E*º=0.996 V), the Te nanosheet could be etched faster in H_2_O_2_ and KMnO_4_ solutions.


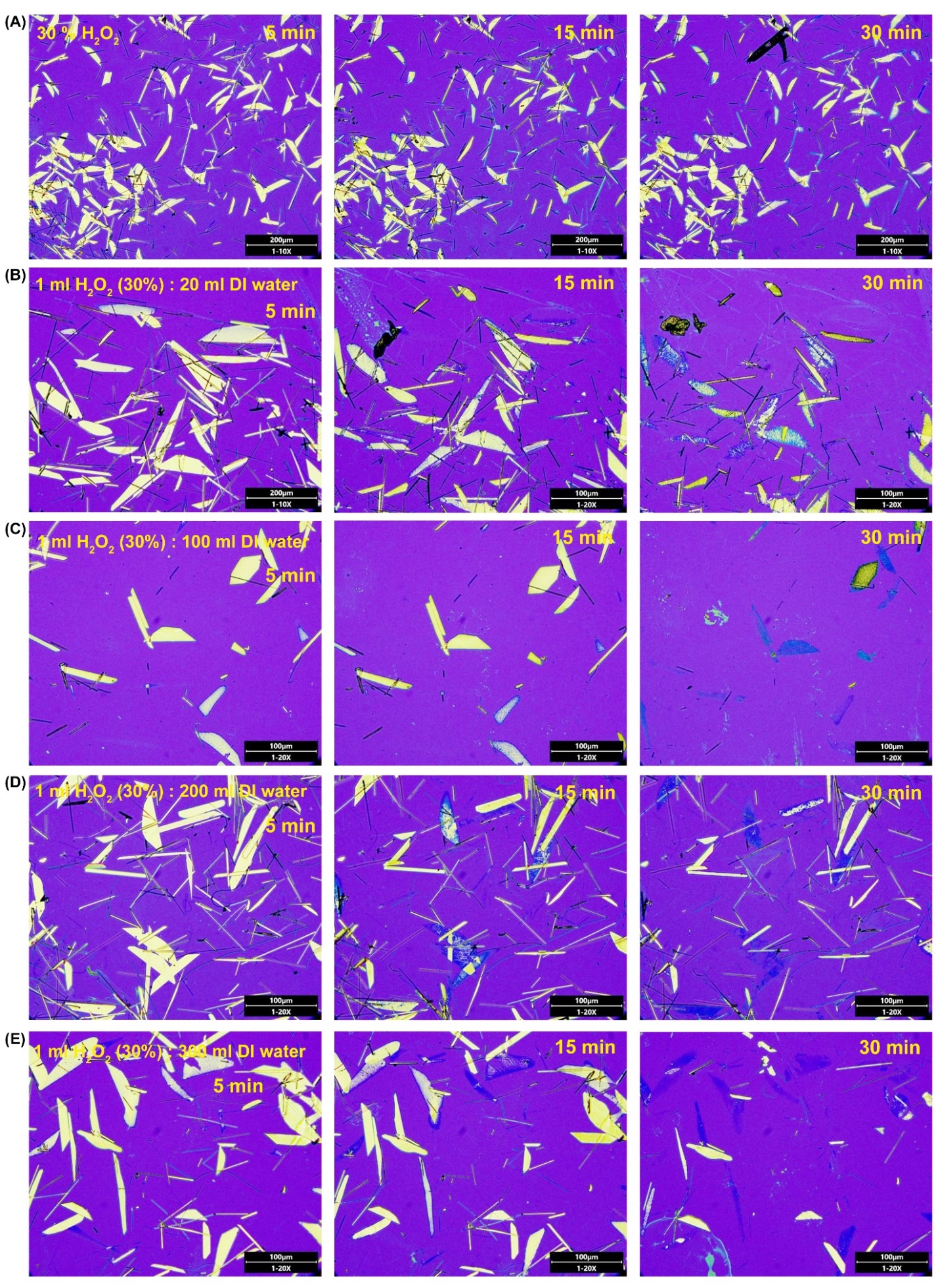


**Supplementary Figure S7.** H_2_O_2_ concentration-dependent thinning effect. Based on our results, the thinning effect is closely relevant to the concentration of H_2_O_2_ solution. Despite the Te nanosheets in the same batch having varying thicknesses, a more dilute H_2_O_2_ solution generally results in a better thinning effect and better control over the material thickness. From **Supplementary Figure S7A**, it can be found that the morphologies of Te flakes are little changed over 30 min immersion in concentrated 30% (w/w) H_2_O_2_. However, ultrathin Te nanosheets could be easily obtained in dilute H_2_O_2_ solution, as shown in **Supplementary Figure S7E**. This phenomenon could be ascribed to the greater extent of ionization of H_2_O_2_ in a more dilution solution.


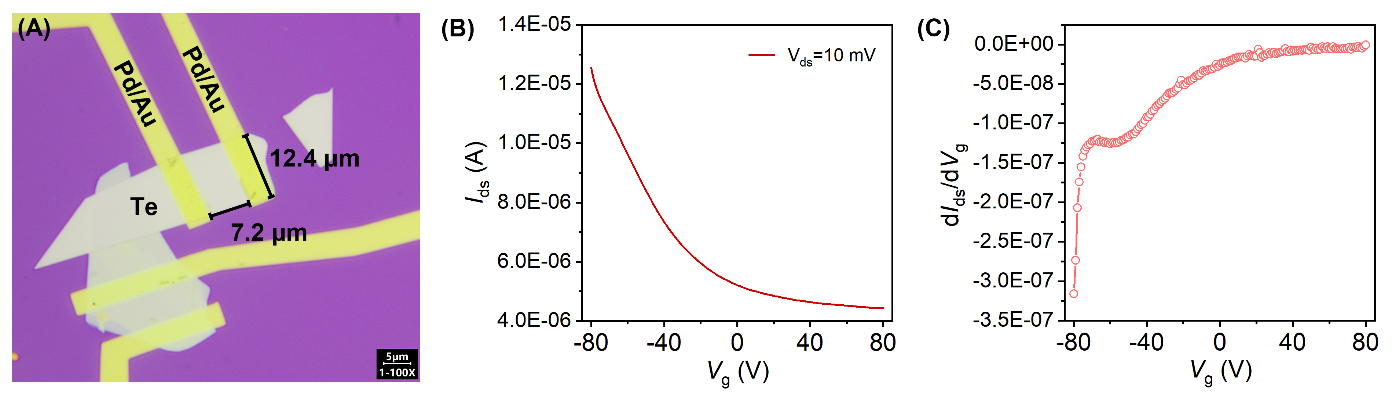


**Supplementary Figure S8.** Carrier mobility calculation for FETs fabricated with as-grown Te. **(A)** Optical image of the FET, the channel length (*L*) and width (*W*) are measured as 7.2 and 12.4 μm, respectively. **(B)** Transfer curve of the FET at a *V*_ds_ of 10 mV. **(C)** Gate-dependent transconductance of the FET, the data are calculated from **(B)**. The thickness of SiO_2_ is ~300 nm. A field-effect hole mobility of ~630 cm^2^V^-1^s^-1^ could be obtained using the equation *μ*=[d*I*_ds_/d*V*_g_] ×[*L*/(*WC*_SiO2_*V*_ds_)].


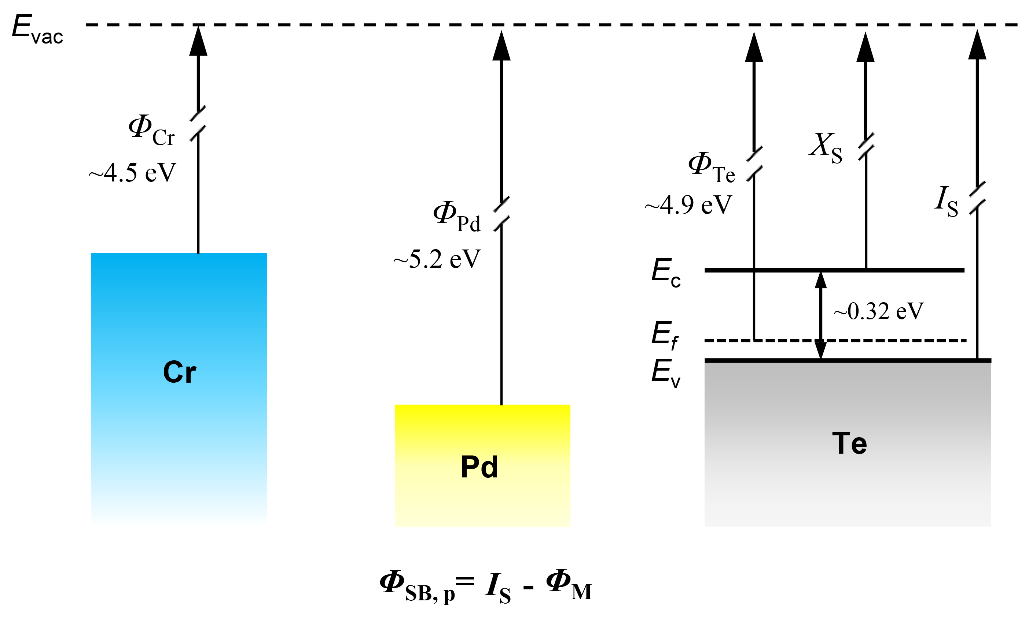


**Supplementary Figure S9.** Schematic energy band profiles for Cr, Pd, and Te. The energy barrier height for hole transport (*Φ*_SB, p_) at the metal-semiconductor interface could be calculated following the Schottky-Mott rule. Previous studies have shown that the work function of bulk Te is ~4.6-4.9 eV (Jałochowski et al., 1972; Michaelson, 1977; Mansingh and Garg, 1984; Velazquez et al., 2012; Choi et al., 2015; Zhang et al., 2015). The Schottky barrier height for hole could be calculated as *Φ*_SB, p_=*I*_S_-*Φ*_M_ according to the Schottky-Mott rule, where *I*_S_ represents the ionization potential of Te, and *Φ*_M_ is the work function of the metal. Therefore, the Schottky barrier height between *p*-Te and high-work-function Pd is supposed to be lower than Cr/*p*-Te, leading to better electrical properties of FETs.


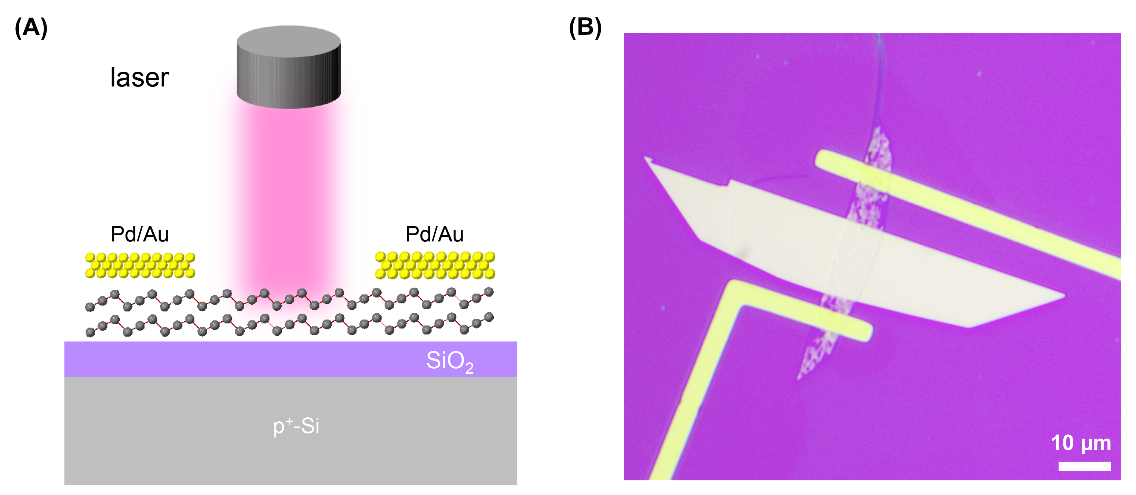


**Supplementary Figure S10.** Schematic **(A)** and optical image **(B)** of the phototransistor device.


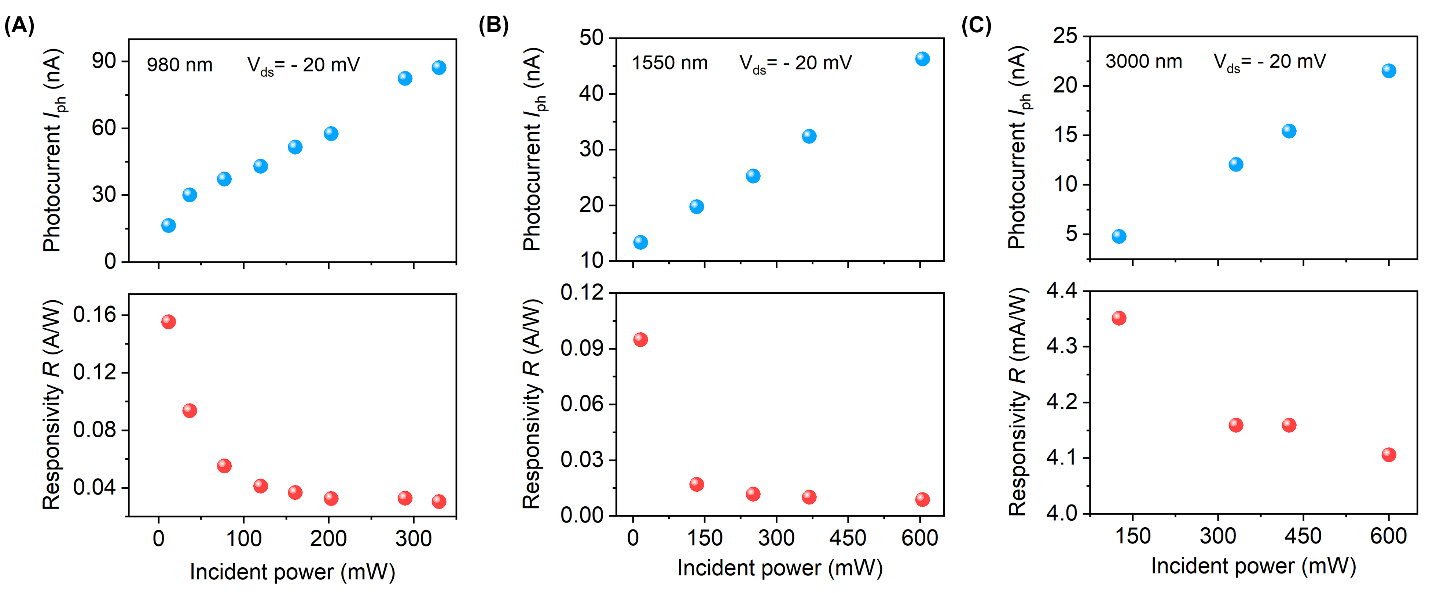


**Supplementary Figure S11.** Illumination power-dependent net photocurrent *I*_ph_ and responsivity *R* for 980 nm **(A)**, 1550 nm **(B)**, and 3000 nm **(C)** laser. The data are extracted from **Figure 5A-C** in the manuscript. The net photocurrent *I*_ph_ is calculated by *I*_ph_=*I*_light_-*I*_dark_, where the *I*_dark_ and *I*_light_ represent the drain current under dark and illumination conditions.


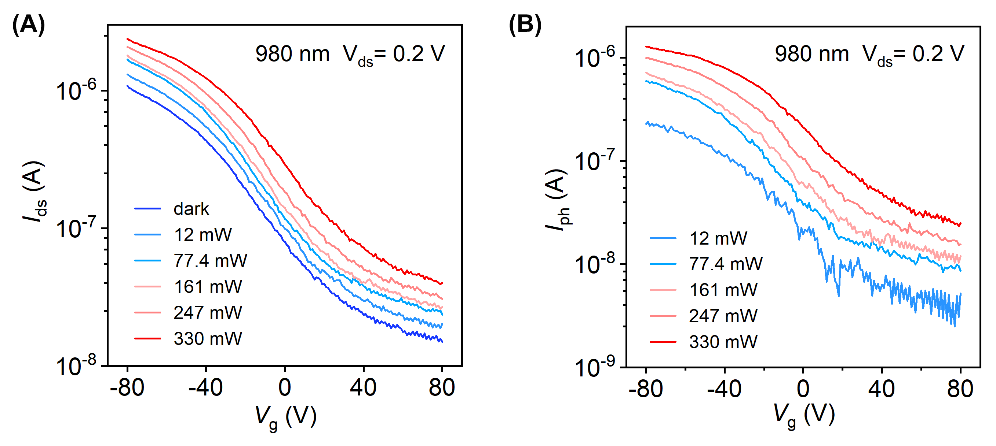


**Supplementary Figure S12.** **(A)** Transfer curves of Te phototransistor under varying 980 nm illumination power. **(B)** Gate-dependent net photocurrent *I*_ph_, the data are extracted from **(A)**.

**Supplementary Note 1:**

Here, we take the transfer curves (*I*_ds_-*V*_g_) of Te phototransistor under 980 nm illumination as an example to calculate the critical parameters, including responsivity *R*, external quantum efficiency *EQE*, and detectivity *D*. The laser spot area is ~0.25 cm^2^ and the effective device area *A* is ~2.18×10^-6^ cm^2^. For the illumination power of 12 mW (corresponding to a power density *P*_in_ of 48 mWcm^-2^), the drain currents under illumination and dark are 1.3073×10^-6^ (*I*_light_) and 1.078×10^-6^ (*I*_dark_) A, respectively, yielding a net photocurrent *I*_ph_ (*I*_ph_=*I*_light_-*I*_dark_) of 2.293×10^-7^ A.

The responsivity is calculated by:

*R*=*I*_ph_/(*P*_in_×*A*) = (2.293×10^-7^ A) / (48 mW/cm^2^ × 2.18×10^-6^ cm^2^) = 2.2 A/W

The external quantum efficiency (*EQE*) is defined as *EQE*=(1.24×*R*)/λ, where λ represents the illumination light wavelength (μm). Accordingly, the *EQE*= (1.24×2.2) / 0.98= 2.8

The calculated detectivity is:

*D =* $R\times\sqrt{\frac{A}{2\times e\times I_{dark}}}$ = 2.2 AW^-1^$\times$ $\sqrt{\frac{2.18 \times{10}^{-6} {cm}^{2}}{2\times1.6\times{10}^{-19} C \times1.078\times{10}^{-6} A}}$ = 5.5×10^9^ cmW^-1^Hz^1/2^


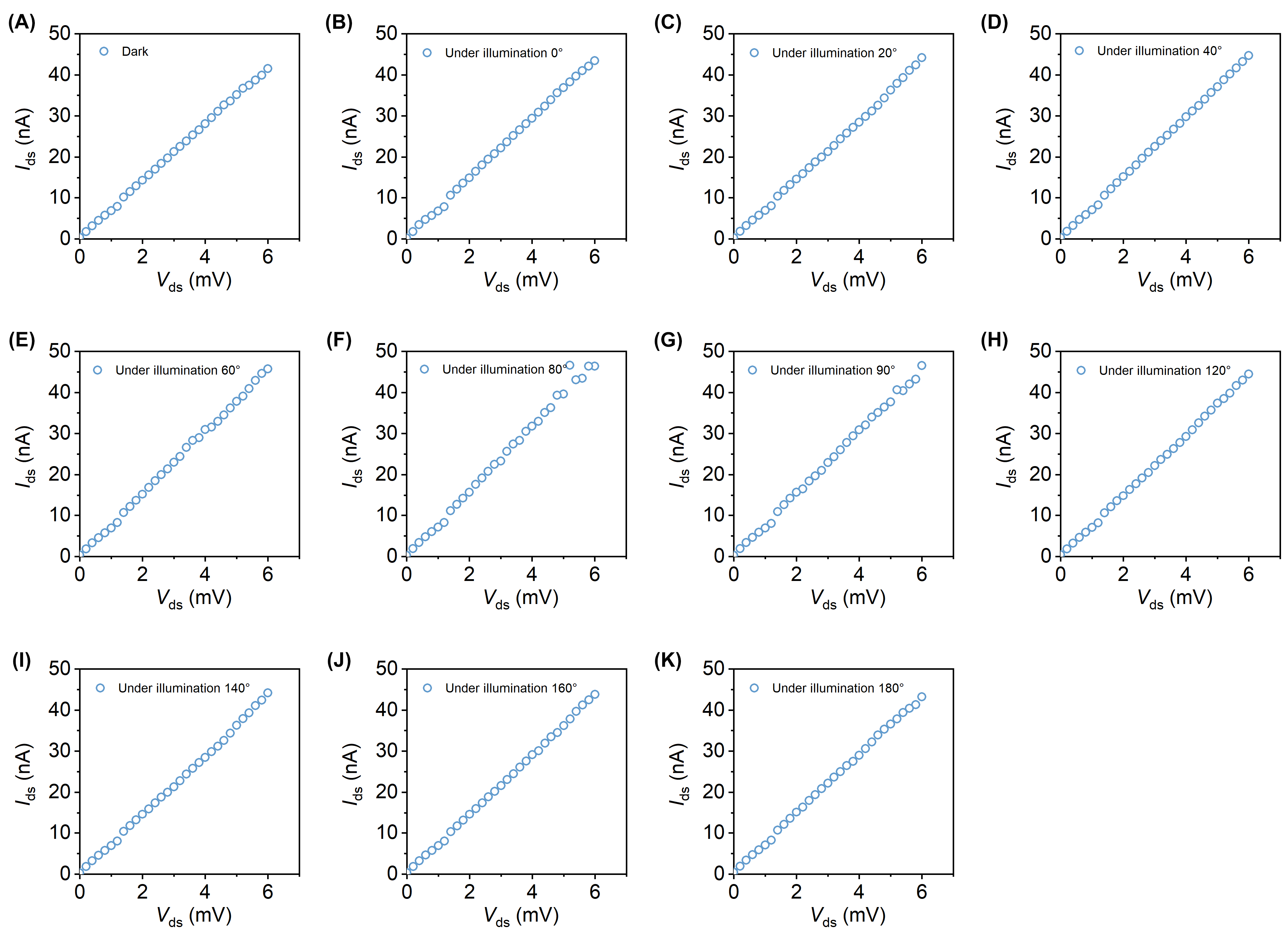


**Supplementary Figure S13.** The *I*_ds_-*V*_ds_ curves of Te transistor at different sample rotation angles under linearly polarized 1.55 μm laser illumination. The laser polarization direction and incident power (~ 360 mW) are kept constant during measurement.

# Supplementary References

Choi, J., Lee, K., Park, C.R., and Kim, H. (2015). Enhanced thermopower in flexible tellurium nanowire films doped using single-walled carbon nanotubes with a rationally designed work function. *Carbon* 94, 577-584. doi: 10.1016/j.carbon.2015.07.043

Jałochowski, M., Mikołajczak, P., and Subotowicz, M. (1972). Measurements of the work function and the fermi level in thin tellurium films. *Phys. Status Solidi A* 14, K135-K137. doi: 10.1002/pssa.2210140250

Mansingh, A., and Garg, A.K. (1984). Electrical properties of ( p)Te‐( p)Si isotype heterojunction. *J. Appl. Phys.* 56, 2315-2322. doi: 10.1063/1.334266

Michaelson, H.B. (1977). The work function of the elements and its periodicity. *J. Appl. Phys.* 48, 4729-4733. doi: 10.1063/1.323539

Velazquez, D., Wisniewski, E.E., Yusof, Z., Harkay, K., Spentzouris, L., and Terry, J. (2012). Kelvin probe studies of cesium telluride photocathode for the AWA photoinjector. *AIP Conf. Proc.* 1507, 780-784. doi: 10.1063/1.4773797

Zhang, Z., Zhang, H., Wu, Y., Zeng, Z., and Hu, Z. (2015). Optimization of the thermopower of antimony telluride thin film by introducing tellurium nanoparticles. *Appl. Phys. A* 118, 1043-1051. doi: 10.1007/s00339-014-8871-8
